# Radiochemistry and comparative in vitro assessment of PSMA-617 labeled with lead-212 (^212^Pb), actinium-225 (^225^Ac), and lutetium-177 (^177^Lu)

**DOI:** 10.1186/s41181-026-00456-w

**Published:** 2026-05-20

**Authors:** Abhijit Bera, Graham Ragland, Yuhan Zhang, Patricia G. Madel, Jasmine B’Lanton, Atchimnaidu Siriki, Chin-Tu Chen, Russell Z. Szmulewitz, Satish K. Chitneni

**Affiliations:** 1 Department of Radiology, The University of Chicago, Chicago, IL USA; 2 Department of Medicine, The University of Chicago, Chicago, IL USA

**Keywords:** PSMA-617, Targeted alpha therapy, ^177^Lu, ^225^Ac, ^212^Pb, Prostate cancer

## Abstract

**Background:**

PSMA-targeted radioligand therapy is a promising approach for the treatment of advanced prostate cancer; however, the clinical efficacy of [^177^Lu]Lu-PSMA-617 (Pluvicto^®^) is limited by the relatively low cytotoxic potency of the β-emitting radionuclide ^177^Lu (t_1/2_: 6.65 d). This has driven high interest in α-emitting radionuclides, such as ^212^Pb (t_1/2_: 10.64 h) and ^225^Ac (t_1/2_: 9.92 d), which deliver high Linear Energy Transfer (LET) and cause more potent tumor cell killing. In this work, we assessed the radiolabeling and in vitro characteristics of ^212^Pb- and ^225^Ac-labeled PSMA-617 compared with [^177^Lu]LuPSMA-617, using two different PSMA-positive prostate cancer cell lines, LNCaP-AR and DU145-PSMA, along with paired negative controls.

**Results:**

The radioligands were synthesized with an isolated radiochemical yield of > 95% for [^177^Lu]Lu-PSMA-617 and about 45% for [^212^Pb]Pb-PSMA-617 and [^225^Ac]Ac-PSMA-617. The molar activity after Sep-Pak purification was about 37.0 MBq/nmol for [^177^Lu]Lu-PSMA-617, 4.4–11.1 MBq/nmol for [^212^Pb]Pb-PSMA-617, and 0.22–0.55 MBq/nmol for [^225^Ac]Ac-PSMA-617. In vitro stability studies in PBS, human serum, and whole blood revealed ≥ 90% stability for [^177^Lu]Lu-PSMA-617 (up to 5 days) and [^212^Pb]Pb-PSMA-617 (24 h), whereas [^225^Ac]Ac-PSMA-617 exhibited ~ 72% stability in PBS, > 94% in serum, and > 86% in whole blood for 5 days. The uptake of [^177^Lu]Lu-PSMA-617 in LNCaP-AR cells was 7.5 ± 0.6%, with about 33% of the cell-bound activity internalized at 4 h. The uptake and internalization were significantly higher in the PSMA-overexpressing cell line DU145-PSMA (31.1 ± 0.6%, 65% internalized). Compared to [^177^Lu]Lu-PSMA-617, [^212^Pb]Pb-PSMA-617 showed about 2-fold higher uptake and increased internalization in LNCaP-AR cells at 4 h (14.2 ± 0.0%, 47.1% internalized), but a similar uptake in DU145-PSMA cells (29.3 ± 0.7%, 62% internalized). In contrast, [^225^Ac]Ac-PSMA-617 exhibited lower uptake in both cell lines, with 1.6 ± 0.2% in LNCaP-AR cells and 4.6 ± 0.2% in DU145-PSMA cells after 4 h incubation. For all 3 radioligands, the uptake could be fully blocked by co-incubation with unlabeled PSMA-617 (300 nM), confirming the specificity of binding to PSMA, and the uptake was minimal in the paired PSMA-negative cell lines CWRR1-EnzR and DU145. In saturation binding assays, the three radioligands exhibited comparable binding affinities (*K*_D_) in LNCaP-AR and DU145-PSMA cell lines, with 5.9 ± 0.7 nM and 2.3 ± 0.3 nM for [^177^Lu]Lu-PSMA-617, 5.7 ± 0.5 nM and 5.8 ± 0.9 nM for [^225^Ac]Ac-PSMA-617, and 2.7 ± 0.5 nM and 8.0 ± 1.1 nM for [^212^Pb]Pb-PSMA-617, respectively.

**Conclusion:**

[^212^Pb]Pb-PSMA-617 showed similar or better uptake in PSMA-positive cell lines compared to [^177^Lu]Lu-PSMA-617. While [^225^Ac]Ac-PSMA-617 also showed selective and specific uptake in the two PSMA-positive cell lines, the uptake levels were substantially lower, likely due to the low molar activities achievable for [^225^Ac]Ac-PSMA-617 compared to [^212^Pb]Pb-PSMA-617 or [^177^Lu]Lu-PSMA-617.

**Supplementary information:**

The online version contains supplementary material available at 10.1186/s41181-026-00456-w.

## Background

Prostate cancer remains a leading cause of cancer-related mortality in men in the United States and worldwide (Kratzer et al. 2025; Sung et al. 2021). Early translational studies demonstrated that PSMA-617, which consists of a DOTA chelator conjugated to the Glu-urea-Lys pharmacophore, exhibits high binding affinity to prostate-specific membrane antigen (PSMA) and efficient internalization into PSMA-positive cells, laying the foundation for PSMA-targeted radioligand therapy (RLT) (Benesova et al. 2015). Subsequent medicinal chemistry efforts and preclinical studies further established PSMA-617 as a robust ligand for labeling with a variety of radionuclides for targeting PSMA expressing prostate cancers (Benesova et al. 2016). Clinical translation of lutetium-177 (^177^Lu, t_1/2_ = 6.65 d) labeled PSMA-617 began shortly thereafter, with early clinical studies demonstrating significant tumor regression, improved overall survival, and favorable safety profiles in men with metastatic castration-resistant prostate cancer (Hofman et al. 2021). The radiopharmaceutical [^177^Lu]Lu-PSMA-617 (Pluvicto) has been approved for the treatment of metastatic castration-resistant prostate cancer following the landmark VISION trial, which demonstrated improved overall survival and radiographic progression-free survival in patients with metastatic castration-resistant prostate cancer (Sartor et al. 2021). Despite its promising therapeutic efficacy, approximately 30–50% of patients exhibit limited or no therapeutic response to [^177^Lu]Lu-PSMA-617 (Kafka et al. 2024; Sartor et al. 2021), highlighting the need for improved therapeutic strategies.

**Fig. 1:**
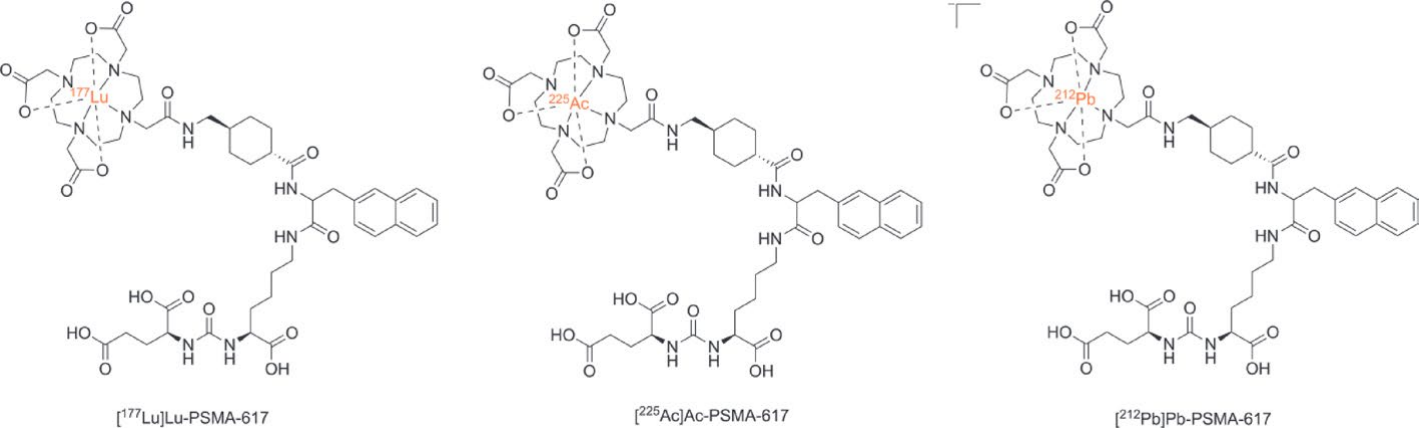
Chemical structures of the three PSMA-617 radioligands synthesized and evaluated in this work.

To address these limitations, α-particle-emitting radionuclides such as actinium-225 (^225^Ac, t_1/2_ = 9.92 d) and lead-212 (^212^Pb, t_1/2_ = 10.64 h) have been investigated for PSMA-targeted RLT. Compared with β-emitting radionuclides, α-emitters such as ^225^Ac deliver high linear energy transfer (LET, ~ 100 keV/µm) radiation with a short path length (50–100 μm), enabling highly localized tumor cell killing through DNA double-strand breaks while minimizing radiation exposure to surrounding healthy tissues (Kratochwil et al. 2016). In line with this, multiple clinical studies have confirmed that ^225^Ac-labeled PSMA-617 can provide therapeutic benefits in men with metastatic castration-resistant prostate cancer, including those refractory to [^177^Lu]Lu-PSMA-617 (Kratochwil et al. 2016; Yadav et al. 2020). Additionally, preclinical studies in mouse models of metastatic prostate cancer demonstrated that early treatment with [^225^Ac]Ac-PSMA-617 prevented the development of liver metastases and significantly improved survival, whereas delayed treatment prolonged survival without significantly reducing tumor burden (Stuparu et al. 2020). Likewise, α-emitters with shorter half-lives, such as ^212^Pb (10.64 h), have also attracted increasing interest for PSMA-targeted RLT. The relatively short half-life of ^212^Pb enables rapid decay and localized dose delivery to tumor cells, potentially reducing α-recoil-driven redistribution of radioactive daughter isotopes (Yong and Brechbiel 2015). In addition, ^212^Pb exhibits favorable radiochemical properties, allowing reproducible synthesis and stable complexation with DOTA-based ligands, while its generator-based production supports scalable clinical availability (Zimmermann 2024). The PSMA-targeting properties of [^212^Pb]Pb-PSMA-617 have been investigated in PSMA-positive C4-2 prostate cancer cells, and its in vivo performance supports further exploration for therapeutic applications (Stenberg et al. 2020). While [^177^Lu]Lu-PSMA-617 has established the clinical role of PSMA-targeted RLT, ^225^Ac- and ^212^Pb-labeled radioligands represent the next generation of RLTs, with the potential to improve therapeutic efficacy and clinical outcomes (Stenberg et al. 2020; Yadav et al. 2020). In this study, we compared the radiolabeling and in vitro properties of ^225^Ac- and ^212^Pb-labeled PSMA-617 with the clinically established [^177^Lu]Lu-PSMA-617 (Fig. 1) using two pairs of PSMA-positive and -negative cell lines.

## Results

### Nonradioactive chemistry

To evaluate the inhibitory potency of the PSMA-617 conjugates, we synthesized nonradioactive reference analogues of [^177^Lu]Lu-PSMA-617 and [^212^Pb]Pb-PSMA-617 by complexing PSMA-617 with lutetium (Lu^3+^) and lead (Pb^2+^) using LuCl_3_ and PbCl_2_, respectively (Figures S1 and S2). The resulting nonradioactive complexes Lu-PSMA-617 and Pb-PSMA-617 were obtained in 37% and 48% yields, respectively, and with > 95% purity after purification using a Sep-Pak C18 cartridge. The identity of the nonradioactive complexes was verified by LC/MS analysis, which showed the expected molecular ion peaks corresponding to the respective nonradioactive PSMA-617 conjugates (Figures S1 and S2). Nonradioactive Ac-PSMA-617 could not be synthesized due to the lack of a stable isotope for actinium.

## Radiochemistry

Radiolabeling of PSMA-617 with ^177^Lu, ^225^Ac, and ^212^Pb was performed following previously published procedures with some modifications (Hooijman et al. 2021; Wurzer et al. 2022). For [^177^Lu]Lu-PSMA-617, the labeling yield increased from 63% with 0.25 nmol precursor (PSMA-617) to 95% with 1 nmol precursor at 37.0 MBq of ^177^LuCl_3_ (Figure S3), providing a molar activity of approximately 37.0 MBq/nmol. Further increase in the amount of precursor (up to 10 nmol) led to ≥ 99% labeling efficiency for the labeled conjugate. In the case of [^225^Ac]Ac-PSMA-617, instant thin-layer chromatography (iTLC) analysis of the crude reaction mixture indicated a labeling efficiency of 80–95% when the reactions were conducted at a 0.2-2 nmol scale (PSMA-617) using 0.37–1.85 MBq of [^225^Ac]AcCl_3_ (Figure S4). However, subsequent purification of the crude reaction mixture using a Sep-Pak revealed that only about 45% of the loaded activity was retained on the column, which was eluted with ethanol and used for in vitro studies. The effective molar activity of [^225^Ac]Ac-PSMA-617 after Sep-Pak purification was 0.35 ± 0.10 MBq/nmol (*n* = 4). Similarly, ^212^Pb-labeling of PSMA-617 was accomplished by reacting 1 nmol PSMA-617 with 18.5 MBq of [^212^Pb]PbCl_2_, eluted from a ^224^Ra/^212^Pb generator in 0.2 M sodium acetate buffer. Sep-Pak purification of the crude mixture after radiolabeling yielded [^212^Pb]Pb-PSMA-617 in 46.3 ± 1.7% (*n* = 4) yield, which was consistent with that determined by the radio-HPLC analysis of the crude reaction mixture (46.5 ± 7.2%) (Figure S5). The molar activity was 7.5 ± 2.3 MBq/nmol (*n* = 4). Radio-HPLC analysis of the purified [^177^Lu]Lu-PSMA-617 and [^212^Pb]Pb-PSMA-617 showed > 98% radiochemical purity, whereas iTLC was used for the [^225^Ac]Ac-PSMA-617 conjugate, which showed a purity of approximately 95%.

## Distribution Coefficient (***D***) Values.

The lipophilicity of the radioconjugates was determined by measuring their distribution coefficient (*D*) values through partitioning between n-octanol and PBS (pH 7.4). The distribution coefficient was calculated as the ratio of radioactivity (CPM) in the octanol phase to that in the aqueous PBS phase and expressed as log*D*_7.4_ values. The measured log*D*_7.4_ values were − 3.29 ± 0.02 (*n* = 4) for [^177^Lu]Lu-PSMA-617, -2.95 ± 0.03 (*n* = 4) for [^225^Ac]Ac-PSMA-617, and − 3.01 ± 0.04 (*n* = 3) for [^212^Pb]Pb-PSMA-617 (Table 1). These results indicate that the three radioligands exhibit comparable lipophilicity, with only minor differences observed among the three PSMA-617-based radioligands.

## In vitro stability studies

The in vitro stability of the three PSMA-617 radioligands were evaluated in PBS, human serum, and human whole blood by incubating the radioligands at 37 °C for up to 120 h. [^177^Lu]Lu-PSMA-617 demonstrated ≥ 90% stability in PBS, human serum, and human whole blood for up to 120 h, as confirmed by iTLC analysis (Figure S6A). [^225^Ac]Ac-PSMA-617 exhibited > 94% stability in human serum over the 120 h incubation period. However, in human whole blood, stability decreased slightly at later time points, from > 95% at 24 h to 86.3% and 89.1% at 96 h and 120 h, respectively. In comparison, stability in PBS decreased more noticeably, reaching 72.3% at 120 h post-incubation (Figure S6B). Because of the short half-life of ^212^Pb, the stability of [^212^Pb]Pb-PSMA-617 was assessed only until 24 h, which revealed ≥ 90% stability in PBS, human serum, and human whole blood (Figure S6C).

## Cell uptake and internalization

**Fig. 2:**
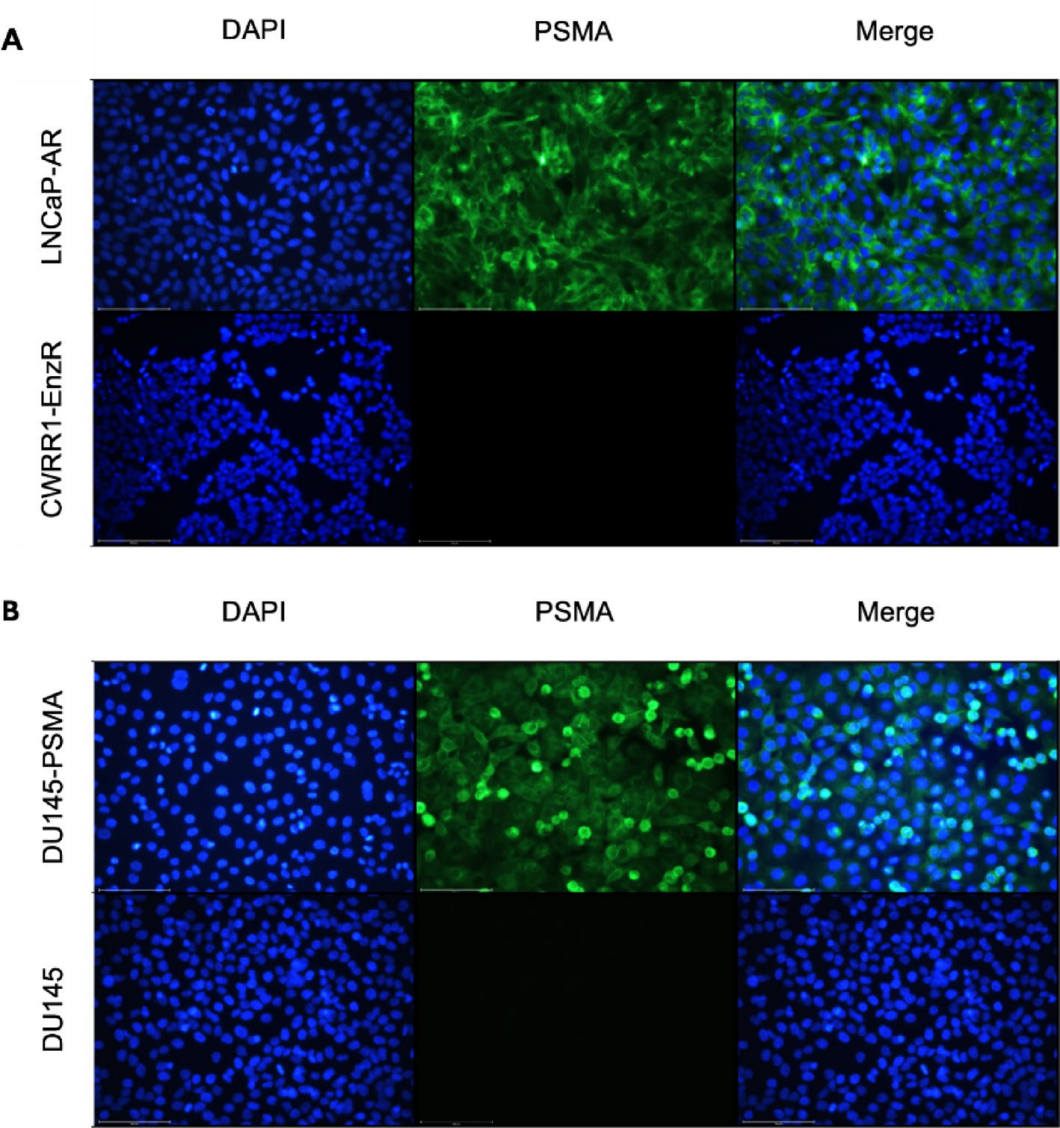
Immunocytochemical imaging of PSMA expression in cell line pairs. Images are paired as (**A**) the LNCaP-AR (PSMA-positive) and CWRR1-EnzR (PSMA-negative) pair, and (**B**) the isogenic DU145-PSMA (PSMA-positive) and DU145 (PSMA-negative) pair

**Fig. 3:**
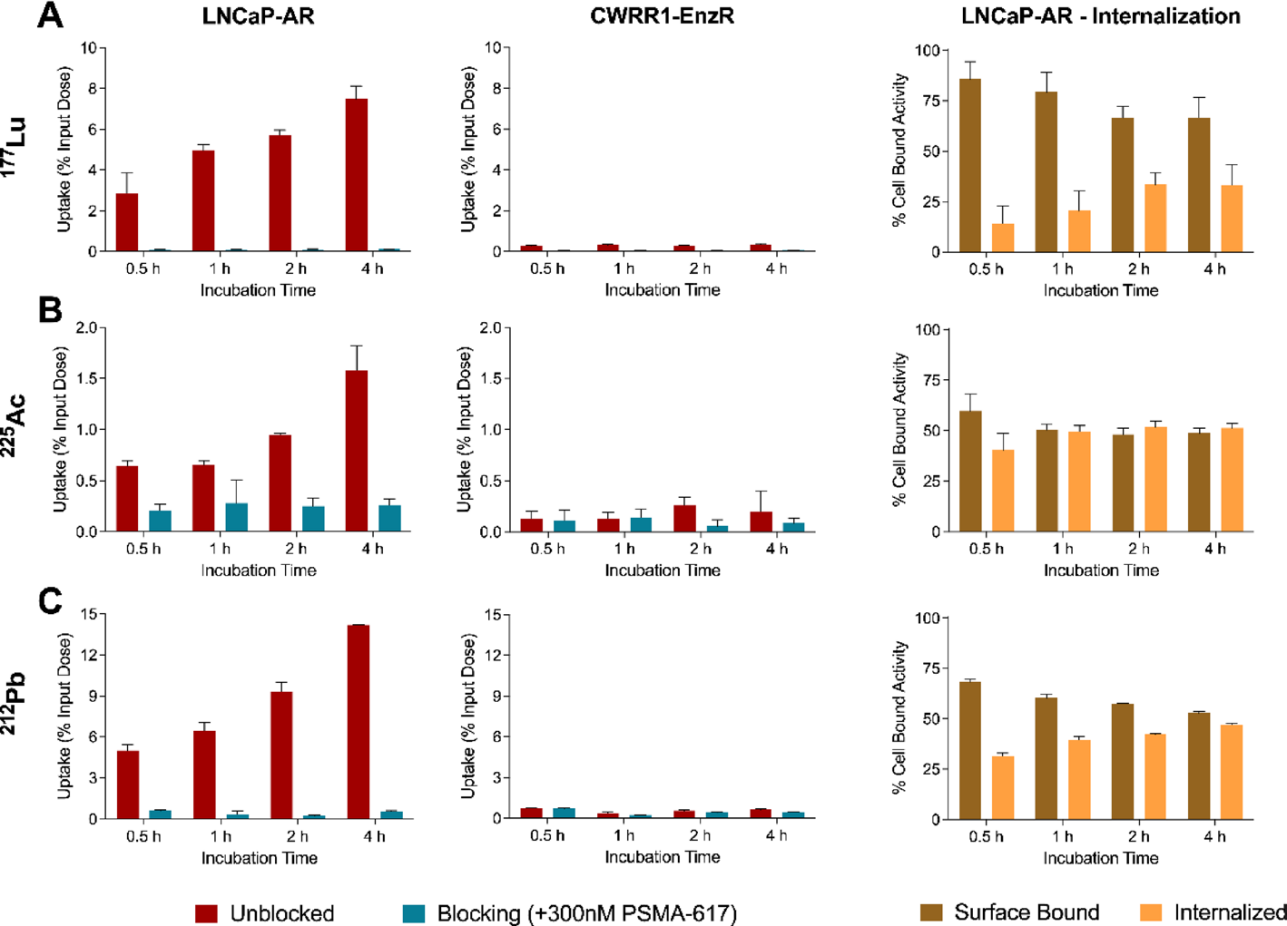
Comparison of cell uptake and internalization in the LNCaP-AR and CWRR1-EnzR cell line pair. Uptake and internalization of (**A**) [^177^Lu]Lu-PSMA-617, (**B**) [^225^Ac]Ac-PSMA-617, and (**C**) [^212^Pb]Pb-PSMA-617 in the PSMA-positive prostate adenocarcinoma cell line LNCaP-AR. For comparison, the uptake of the three radioligands in the PSMA-negative CWRR1-EnzR cell line is also shown (center column). Uptake is presented on different scales (y-axis) for each radioligand

The uptake, specificity, selectivity, and internalization rate of [^177^Lu]Lu-PSMA-617, [^225^Ac]Ac-PSMA-617, and [^212^Pb]Pb-PSMA-617 were compared in two pairs of PSMA-positive and negative prostate cancer cell lines: LNCaP-AR (PSMA-positive) and CWRR1-EnzR (PSMA-negative), and DU145-PSMA (PSMA-positive) and DU145 (PSMA-negative). Immunocytochemical staining followed by fluorescence imaging confirmed high PSMA expression in the two PSMA-positive cell lines LNCaP-AR and DU145-PSMA, with minimal expression in the respective control cell lines CWRR1-EnzR and DU145 (Fig. 2). Cell uptake studies showed that the uptake of [^177^Lu]Lu-PSMA-617 in the LNCaP-AR cell line increased over time, from 2.86 ± 1.02% of added activity at 0.5 h post-incubation to 7.48 ± 0.65% at 4 h (Fig. 3A). Uptake in the CWRR1-EnzR1 cell line was minimal at all time points, with 0.34 ± 0.03% uptake at 4 h. Co-incubation with excess unlabeled PSMA-617 (300 nM) blocked uptake by > 95%, limiting it to 0.12 ± 0.01% at 4 h in LNCaP-AR cells, confirming the specificity and selectivity of [^177^Lu]Lu-PSMA-617 binding in the LNCaP-AR cell line. In contrast to [^177^Lu]Lu-PSMA-617, uptake of [^225^Ac]Ac-PSMA-617 in LNCaP-AR cells was low, with 0.64 ± 0.05% of added activity at 0.5 h but increasing to 1.58 ± 0.24% at 4 h (Fig. 3B). Co-incubation with excess unlabeled PSMA-617 (300 nM) reduced the uptake of [^225^Ac]Ac-PSMA-617 to 0.26 ± 0.06% at 4 h, confirming the specificity. Uptake in the PSMA-negative CWRR1-EnzR cells remained minimal at all time points, with 0.20 ± 0.05% uptake at 4 h, which reduced to 0.09 ± 0.04% with blocking. Similar to the above two radioligands, uptake of [^212^Pb]Pb-PSMA-617 in LNCaP-AR cells increased over time, reaching 14.19 ± 0.03% at 4 h compared to 5.01 ± 0.45% at 0.5 h (Fig. 3C). Co-incubation with excess unlabeled PSMA-617 (300 nM) reduced uptake to 0.58 ± 0.05% at 4 h, and uptake of [^212^Pb]Pb-PSMA-617 in the PSMA-negative CWRR1-EnzR cells remained minimal at all time points, with 0.65 ± 0.03% at 4 h. Additionally, for [^177^Lu]Lu-PSMA-617 and [^212^Pb]Pb-PSMA-617, the fraction of internalized activity, calculated as the percentage of total uptake in cells, increased with time, from 14.2 ± 8.6% at 0.5 h to 33.3 ± 10.0% at 4 h for [^177^Lu]Lu-PSMA-617 (Fig. 3A) and from 31.6 ± 1.5% at 0.5 h to 47.1 ± 0.9% at 4 h for [^212^Pb]Pb-PSMA-617 (Fig. 3C). Compared to these, [^225^Ac]Ac-PSMA-617 exhibited less increase in internalization over time from 40.3 ± 8.4% at 0.5 h to 51.2 ± 2.4% by 4 h (Fig. 3B).

**Fig. 4:**
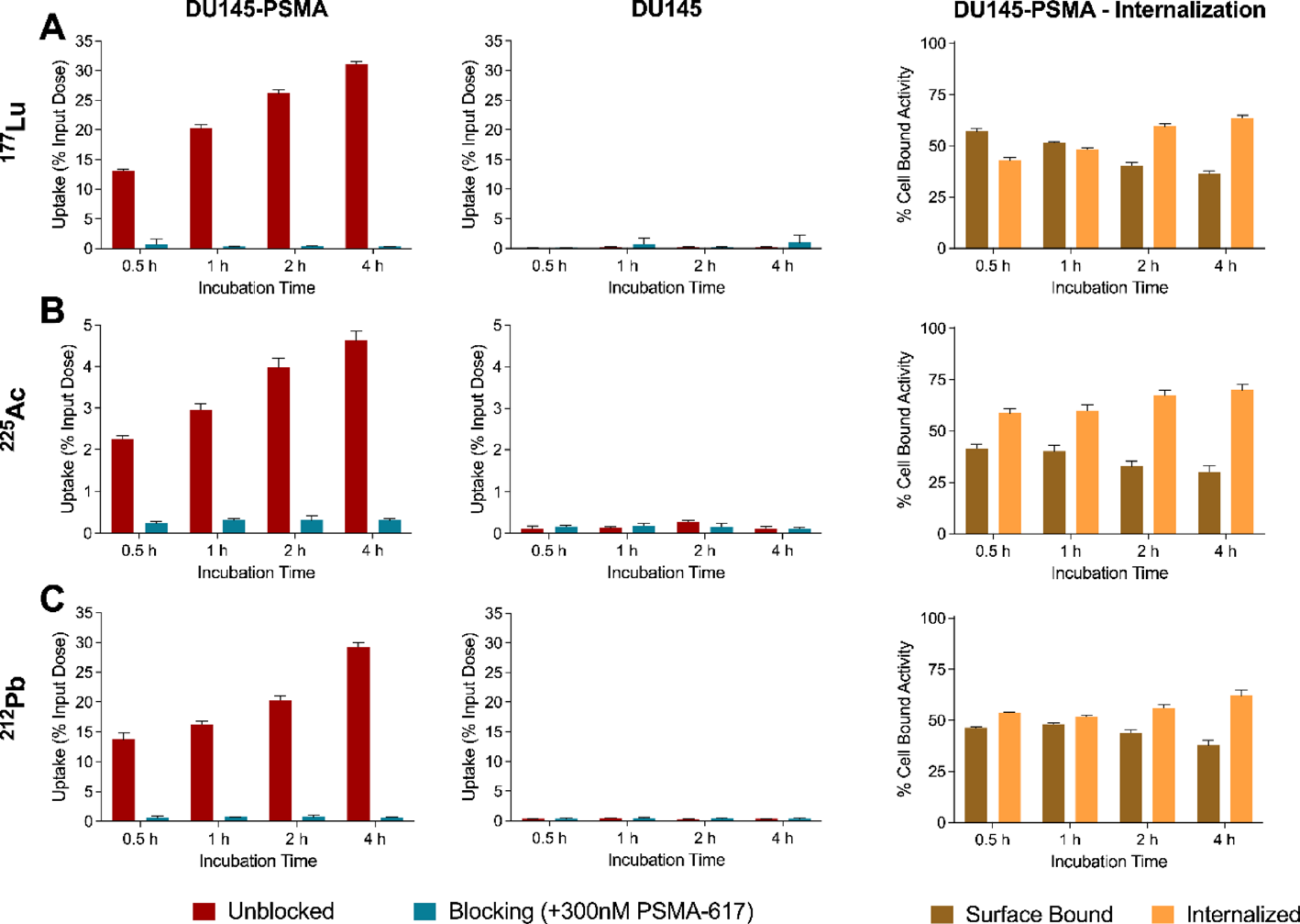
Comparison of cell uptake and internalization in the DU145-PSMA and DU145 cell line pair. Uptake and internalization of (**A**) [^177^Lu]Lu-PSMA-617, (**B**) [^225^Ac]Ac-PSMA-617, and (**C**) [^212^Pb]Pb-PSMA-617 in the PSMA overexpressing DU145-PSMA cell line. For comparison, the uptake of the three radioligands in the PSMA-negative parental DU145 cell line is also shown (center column). Uptake is presented on a different scale (y-axis) for [^225^Ac]Ac-PSMA-617

Cell uptake and internalization of the three radioligands were also evaluated in a second pair of isogenic cell lines, a PSMA-overexpressing DU145-PSMA cell line (courtesy of Novartis Institutes for BioMedical Research) and the parental DU145 cell line (PSMA-negative). The results from these studies are consistent with the uptake trends observed for the three radioligands in the LNCaP-AR cell line, but with significantly higher uptake (by 2-3-fold) than in the LNCaP-AR cell line. The uptake of [^177^Lu]Lu-PSMA-617 in DU145-PSMA cells at 4 h was 31.10 ± 0.64% compared to 0.29 ± 0.02% with blocking (300 nM PSMA-617) and 0.22 ± 0.03% in the parental DU145 cells (Fig. 4A). Consistent with results from the LNCaP-AR cell line, [^225^Ac]Ac-PSMA-617 exhibited lower uptake in the DU145-PSMA cells; nonetheless, the uptake increased over time, from 2.26 ± 0.08% at 0.5 h to 4.63 ± 0.18% at 4 h (Fig. 4B). Co-incubation of cells with an excess of PSMA-617 (300 nM) reduced the uptake to 0.31 ± 0.01% at 4 h, and the uptake in control DU145 cells remained minimal (0.12 ± 0.05%). In contrast, [^212^Pb]Pb-PSMA-617 exhibited a high uptake that is comparable to [^177^Lu]Lu-PSMA-617 in DU145-PSMA cells. Uptake at 0.5 h was 13.74 ± 1.07%, increasing with time to 29.26 ± 0.69% at 4 h. For comparison, uptake in the parental DU145 cell line was 0.32 ± 0.02%, and 0.70 ± 0.04% with blocking (300 nM PSMA-617) at 4 h. (Fig. 4C). For all three radioligands, > 60% of uptake was found to be internalized in DU145-PSMA cells at 4 h, with the remaining activity (< 40%) being cell-surface bound (Fig. 4). Taken together, these results demonstrate high PSMA-specific uptake of the three PSMA-617 radioconjugates in PSMA-expressing cells, with minimal uptake observed in PSMA-negative controls. However, the magnitude of uptake was lower for [^225^Ac]Ac-PSMA-617 relative to [^212^Pb]Pb-PSMA-617 and the reference [^177^Lu]Lu-PSMA-617 in both the cell lines (Figure S7).

## Inhibitory potency (IC_50_) assays

**Fig. 5:**
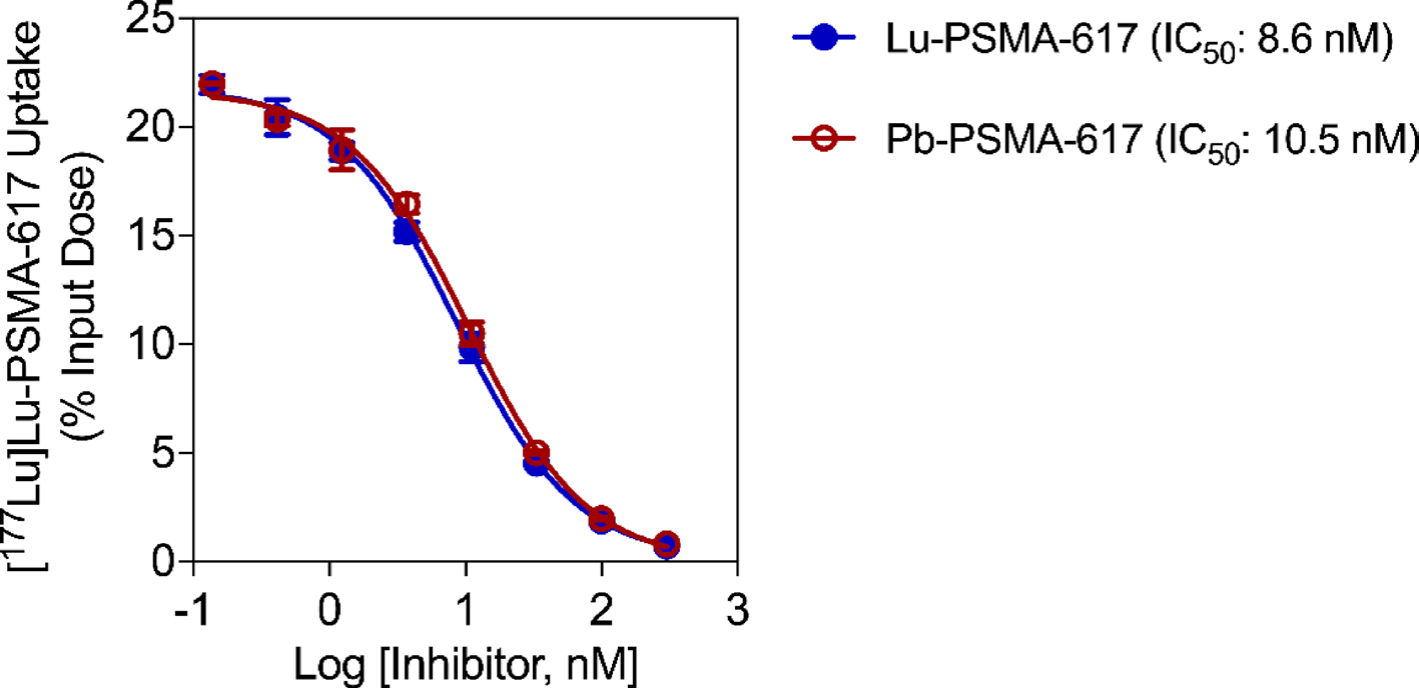
Determination of inhibitory potency of nonradioactive analogues against PSMA using [^177^Lu]Lu-PSMA-617. Competitive inhibition of [^177^Lu]Lu-PSMA-617 uptake in DU145-PSMA cells by the nonradioactive Lu-PSMA-617 or Pb-PSMA-617, with the corresponding half-maximal inhibitory value (IC_50_) shown in parentheses

The inhibitory potencies (IC_50_) of the nonradioactive analogues Lu-PSMA-617 and Pb-PSMA-617 were evaluated by a competitive inhibition assay using [^177^Lu]Lu-PSMA-617 in DU145-PSMA cells. Cells were incubated with [^177^Lu]Lu-PSMA-617 in the presence of increasing concentrations of Lu-PSMA-617 or Pb-PSMA-617 (0.14–300 nM). Cell uptake, presented as the percentage of added activity, was assessed at each concentration to determine the half-maximal inhibitory concentration value (IC_50_) for the two nonradioactive PSMA-617 inhibitors. The results demonstrated concentration-dependent inhibition of the radiotracer uptake in the DU145-PSMA cell line. The inhibitory potency (IC_50_) values derived from these assays were 8.6 nM for Lu-PSMA-617 and 10.5 nM for Pb-PSMA-617 (Fig. 5), indicating similar inhibitory potency for the two PSMA-617 conjugates toward PSMA.

### Binding affinity studies

**Fig. 6:**
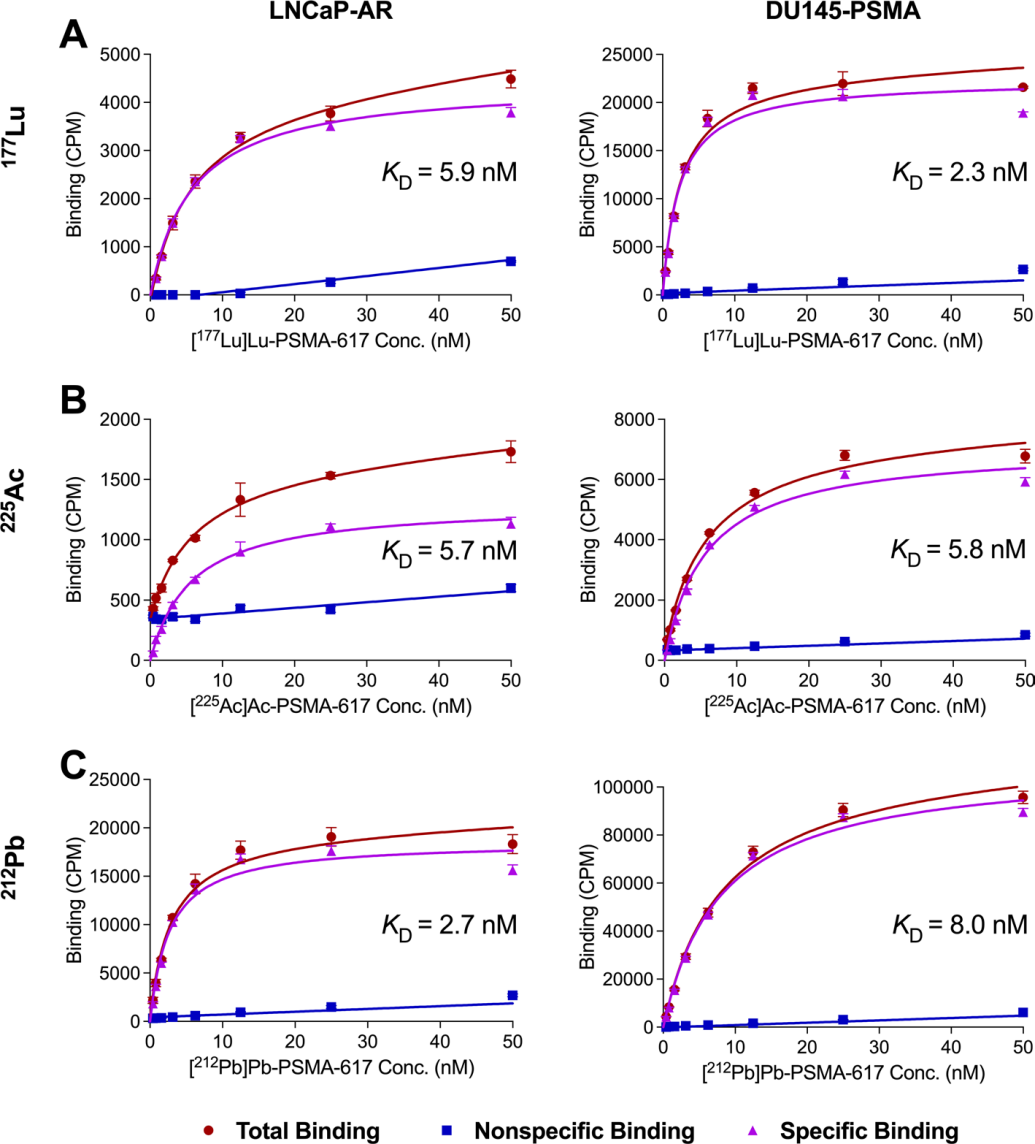
Determination of binding affinity of the three radioligands in the LNCaP-AR and DU-145-PSMA cell lines. Saturation binding of (**A**) [^177^Lu]Lu-PSMA-617, (**B**) [^225^Ac]Ac-PSMA-617, and (**C**) [^212^Pb]Pb-PSMA-617 in the PSMA-positive LNCaP-AR and DU145-PSMA cell lines. Equilibrium dissociation constant (*K*_D_) values determined from these assays are shown for the corresponding radioligand. For each radioligand concentration, nonspecific binding was determined by blocking the uptake using 300 nM PSMA-617

The binding affinities (*K*_D_) of the three radioligands for PSMA were evaluated using the LNCaP-AR and DU145-PSMA cell lines by conducting saturation binding assays as described previously (Chitneni et al. 2018). Cells were incubated with increasing concentrations of ^177^Lu-, ^225^Ac-, or ^212^Pb-labeled PSMA-617 (0.4–50.0 nM) for 2 h, and the cell-bound activity was assessed by gamma counting. For each concentration, nonspecific binding was measured in parallel by co-incubating cells with an excess of unlabeled PSMA-617 (300 nM). Specific binding data were generated by subtracting nonspecific binding from total binding for each concentration, and nonlinear regression analysis was conducted in GraphPad Prism (Fig. 6). Equilibrium dissociation constant values (*K*_D_), determined as the concentration needed to achieve half-maximum binding at equilibrium, were derived from these assays for the three radioligands in the two PSMA-positive cell lines. The *K*_D_ values determined from these assays in LNCaP-AR and DU145-PSMA cells were 5.9 ± 0.7 nM and 2.3 ± 0.3 nM for [^177^Lu]Lu-PSMA-617 (Fig. 6A), compared to 5.7 ± 0.5 nM and 5.8 ± 0.9 nM for [^225^Ac]Ac-PSMA-617 (Fig. 6B) and 2.7 ± 0.5 nM and 8.0 ± 1.1 nM for [^212^Pb]Pb-PSMA-617 (Fig. 6C), respectively.

**Table 1:** Overview of in vitro properties of the three PSMA-617 radioligands synthesized in this work, [^177^Lu]Lu-PSMA-617, [^225^Ac]Ac-PSMA-617, and [^212^Pb]Pb-PSMA-617

Radioligand	Lipophilicity (log D)	Molar Activity (per nmol)	Cell Uptake (PSMA+, 4 h)		Binding Affinity (K_D_)	
			LNCaP-AR	DU145-PSMA	LNCaP-AR	DU145-PSMA
[^177^Lu]Lu-PSMA-617	−3.29	37.0–74.0 MBq	7.5 ± 0.6%	31.1 ± 0.6%	5.9 nM	2.3 nM
[^225^Ac]Ac-PSMA-617	−2.95	0.22–0.55 MBq	1.6 ± 0.2%	4.6 ± 0.2%	5.7 nM	5.8 nM
[^212^Pb]Pb-PSMA-617	−3.01	4.4–11.1 MBq	14.2 ± 0.0%	29.3 ± 0.7%	2.7 nM	8.0 nM

## Discussion

Our radiolabeling experiments of PSMA-617 with the β-emitter ^177^Lu and the α-emitters ^225^Ac and ^212^Pb revealed important differences in radiolabeling characteristics and radiochemical yields for the corresponding radionuclides. Unlike ^177^Lu, radio-HPLC-based QC analysis is less informative for ^225^Ac due to its complex decay scheme and low levels of radioactivity employed, necessitating the use of iTLC-based methods to determine radiolabeling yields and/or purity of the labeled compounds. Although our iTLC analysis of the crude reaction mixture consistently showed > 95% labeling with less free ^225^Ac (Figure S4), subsequent Sep-Pak purification typically retained only ~ 45% of the loaded activity, with the remainder of the activity either unretained or washed off with the water rinse. Careful assessment of the iTLC chromatograms of the crude reaction mixture and the unretained activity fractions indicated the presence of a labeled species migrating at the front of the [^225^Ac]Ac-PSMA-617 peak on the iTLC (at 40–50 mm, Figure S4). Thus, for all subsequent studies and in vitro evaluation of [^225^Ac]Ac-PSMA-617, the reaction mixture was purified by Sep-Pak, and the radioligand was freshly synthesized on the day of each in vitro experiment. Additionally, all cell uptake studies were conducted in the presence of DTPA (0.1 mg/mL) in the incubation mixture to chelate any free ^225^Ac or daughter isotopes, although comparable results were obtained without DTPA as well in select experiments (Figure S8). In contrast, radiolabeling of PSMA-617 with ^212^Pb proved more straightforward. However, despite optimization efforts and increasing the precursor amount (PSMA-617), the RCY could not be improved beyond ~ 50%. Nonetheless, the molar activities we obtained for [^212^Pb]Pb-PSMA-617 in the present study proved sufficient (4.4–11.1 MBq/nmol) for efficient cell uptake and internalization and yielded similar or higher uptake than [^177^Lu]Lu-PSMA-617 in the two PSMA-positive cell lines we evaluated (Figs. 3 and 4).

Across the three radioligands, lipophilicity values were comparable (Table 1) and reflect the overall hydrophilicity of the molecule (PSMA-617), suggesting that substitution of ^177^Lu in [^177^Lu]Lu-PSMA-617 with ^225^Ac or ^212^Pb may not significantly alter the pharmacokinetic properties of the resulting radioconjugates in vivo. However, radionuclide-specific differences in in vivo stability and the impact of progeny isotopes on the pharmacokinetics of the ⍺-emitting radioligands could not be ruled out based on their log*D*. In vitro stability studies demonstrated excellent radiochemical stability for [^177^Lu]Lu-PSMA-617 and [^225^Ac]Ac-PSMA-617, with ≥ 90% stability in PBS, human serum, and human whole blood until 72 h, although the study was extended to 120 h. In view of the short half-life of ^212^Pb (10.6 h), the stability of [^212^Pb]Pb-PSMA-617 was evaluated only for 24 h, which showed ≥ 90% stability of the labeled conjugate.

Comparative cell uptake studies in the two PSMA-expressing prostate cancer cell lines revealed distinct trends for the three isotopes or the radioligands. In LNCaP-AR cells, [^212^Pb]Pb-PSMA-617 exhibited about 2-fold higher uptake than [^177^Lu]Lu-PSMA-617, but similar levels of uptake were noted for both radioligands in the DU145-PSMA cell line. Despite the differences in the total uptake levels, all three radioligands exhibited a higher uptake and internalized fraction in the PSMA overexpressing cell line DU145-PSMA than in LNCaP-AR at 4 h post-incubation. Few studies have reported the radiosynthesis and in vivo evaluation of [^225^Ac]Ac-PSMA-617 (Busslinger et al. 2022; Stuparu et al. 2020); however, systematic evaluation of the labeled conjugate using in vitro models and head-to-head comparison with the established PSMA-targeted radioligand [^177^Lu]Lu-PSMA-617 and/or [^212^Pb]Pb-PSMA-617 is largely lacking. We believe that the lower uptake of [^225^Ac]Ac-PSMA-617 compared to [^177^Lu]Lu-PSMA-617 and [^212^Pb]Pb-PSMA-617 in the two PSMA-positive cell lines was primarily due to the low molar activity of the radioligand, resulting in competitive inhibition of the radioligand by the unlabeled PSMA-617 in the final product. Although differences in cell numbers and cell models preclude direct comparison of our data with literature reports, Brusslinger et al. have noted a comparable uptake for [^225^Ac]Ac-PSMA-617 (63 ± 5%) with that of [^177^Lu]Lu-PSMA-617 (60 ± 5%), with a slightly higher internalized fraction for [^225^Ac]Ac-PSMA-617, at 4 h post-incubation in the PSMA overexpressing cell line PC-3 PIP (Busslinger et al. 2022). Concerning prior reports on the cell uptake of [^212^Pb]Pb-PSMA-617, Stenberg et al. have reported a cell uptake value of 33.2 ± 9.7% (%AA/10^6^ cells) and an internalized fraction of 23.3 ± 4.7% in C4-2 cells at 45 min, which is comparable to the uptake observed for [^212^Pb]Pb-PSMA-617 in LNCaP-AR cells in the present study when normalized to the same cell number (150,000 per well) (Stenberg et al. 2020). In the present study, our initial attempts to achieve a molar activity of at least 3.7 MBq/nmol for [^225^Ac]Ac-PSMA-617, on par with [^212^Pb]Pb-PSMA-617, were unsuccessful. In contrast, an acceptable molar activity was achieved for [^212^Pb]Pb-PSMA-617. The similar or higher uptake of [^212^Pb]Pb-PSMA-617 compared to [^177^Lu]Lu-PSMA-617 in our cell uptake studies indicates a desired molar activity of ≥ 3.7 MBq/nmol for efficient uptake of PSMA-617-based radioligands in PSMA expressing prostate cancer cells. Minimal uptake of the three radioligands in the PSMA-negative cell lines (CWRR1-EnzR and DU145) and efficient blocking with excess PSMA-617 in the two PSMA-positive cell lines confirms the specificity of the three radioligands to PSMA and the PSMA-mediated internalization of the radioligands in our cell models.

Although molar activities, uptake, and internalization rates varied between cell lines, saturation binding assays demonstrated comparable binding affinity values (*K*_*D*_) in the nM range for ^177^Lu-, ^225^Ac-, and ^212^Pb-labeled PSMA-617 in the two PSMA-positive cell lines LNCaP-AR and DU145-PSMA (Fig. 6). Our results strongly suggest that the change of radioisotope did not affect the binding affinity of labeled PSMA-617 in PSMA-expressing cells. Additionally, the observed binding affinity values are consistent with those reported in the literature, e.g., a *K*_D_ of about 4.4 nM for [^177^Lu]Lu-PSMA-617 to LNCaP cells (Peng et al. 2025), 11 nM for [^225^Ac]Ac-PSMA-617 to PC-3 PIP cells (Busslinger et al. 2022), and about 11.1 nM for [^212^Pb]Pb-PSMA-617 to C4-2 cells (Stenberg et al. 2020).

Collectively, our results indicate the suitability of [^225^Ac]Ac-PSMA-617 and [^212^Pb]Pb-PSMA-617 for further evaluation using in vivo models of PSMA-expressing prostate cancer in comparison with [^177^Lu]Lu-PSMA-617. Although it may not be possible to achieve high molar activity for [^225^Ac]Ac-PSMA-617 similar to that for ^177^Lu- or ^212^Pb-labeled PSMA-617, it remains to be tested if the reduced cell uptake from in vitro models translates to lower tumor uptake (e.g., percentage injected dose per gram (% ID/g)) in vivo compared to [^177^Lu]Lu-PSMA-617 or [^212^Pb]Pb-PSMA-617. To that end, our future studies will focus on extending these in vitro studies to in vivo by directly comparing tumor uptake of the three radioligands and the therapeutic efficacy of the ⍺-emitting PSMA-617 radioligands with [^177^Lu]Lu-PSMA-617 in the same tumor models.

## Conclusion

This study was designed to directly compare radiolabeling and in vitro characteristics of PSMA-617 labeled with α- and β-emitting radionuclides as a prelude to in vivo comparisons in the same tumor models. In this study, we successfully optimized radiolabeling protocols for [^225^Ac]Ac-PSMA-617 and [^212^Pb]Pb-PSMA-617, achieving high radiochemical purity and in vitro stability suitable for in vivo evaluation. Both the α-emitting radioligands demonstrated selective uptake, specificity, and efficient internalization in two different PSMA-expressing prostate cancer cell lines. Notably, [^212^Pb]Pb-PSMA-617 exhibited an in vitro uptake comparable to or exceeding that of [^177^Lu]Lu-PSMA-617 in PSMA-positive cells, highlighting its potential as a targeted α-therapy agent for PSMA-expressing prostate cancers. Although [^225^Ac]Ac-PSMA-617 exhibited PSMA-specific uptake in the two cell lines we evaluated, the uptake levels were substantially lower, most likely due to the low molar activities achievable for ^225^Ac-labeling versus ^212^Pb- and ^177^Lu-labeling. Collectively, the results from this study support further evaluation of [^212^Pb]Pb-PSMA-617 and [^225^Ac]Ac-PSMA-617 to directly compare their tumor uptake and therapeutic efficacy in prostate cancer models for targeted α-therapy in comparison with [^177^Lu]Lu-PSMA-617.

### Experimental section

#### General methods

All reagents and solvents were purchased from Fisher Scientific, MilliporeSigma, or Ambeed and used as received unless otherwise noted. PSMA-617 was supplied by Novartis Institutes for BioMedical Research (NIBR, Basel, Switzerland). TraceSELECT™ water was purchased from Honeywell, and Chelex^®^ 100 resin was obtained from Bio-Rad. All buffers and reagents used in this work were prepared using Chelex-treated water unless specified. Nonradioactive complexes were analyzed using an expression-L compact mass spectrometer coupled to a high-performance liquid chromatography (HPLC) system equipped with a UV detector and operated using Mass Express software (Advion Interchim Scientific, Ithaca, NY). The following method was used for the analysis: Agilent Poroshell 120 C18 column (3 × 50 mm); mobile phases: A = water with 0.1% trifluoroacetic acid (TFA), B = acetonitrile (MeCN) with 0.1% TFA; flow rate: 0.3 mL/min; gradient conditions: 0–0.3 min, 5% B; 0.3–9 min, 5→95% B; 9–9.5 min, 95→5% B.

All three isotopes, ^177^Lu, ^225^Ac, and ^212^Pb, were purchased through the U.S. Department of Energy’s National Isotope Development Center (NIDC). ^177^Lu was supplied as ^177^LuCl_3_ in 0.04 M HCl. ^225^Ac was supplied as dry ^225^AcNO_3_. ^212^Pb was obtained in the form of a ^224^Ra/^212^Pb generator and eluted following the instructions provided with the generator. Briefly, a Pb resin QML cartridge (20–50 μm, Eichrom Technologies, Lisle, IL) was preconditioned with 2 M HCl (1 mL) and connected to the outlet of the generator. Then 1 mL of 2 M HCl was passed through the inlet line of the generator to elute and capture ^212^Pb on the Pb cartridge. The tubing was purged with air, after which the Pb cartridge was disconnected from the generator. The generator was then rinsed with 2 mL of water and was left with water for storage until the next elution (the next day). The Pb resin cartridge, containing ^212^Pb, was then rinsed with water (1 mL) to remove residual acid. The cartridge was reversed, and ^212^Pb was eluted by passing 0.2-1.0 M sodium acetate (NaOAc) solution (0.5–1 mL), which was used directly for radiolabeling reactions without further modification. HPLC analysis of radioactive samples (^177^Lu, ^212^Pb) was conducted using an Agilent 1260 Infinity II quaternary pump system coupled to an Agilent 1260 Infinity II variable-wavelength UV detector and a Dual Scan-RAM radio-TLC and radio-HPLC detector (LabLogic, Chantilly, VA). The following method was used for the HPLC analysis: Kinetex^®^ 5 μm EVO C18 column (4.6 × 150 mm, 100 Å); mobile phases: A = water with 0.1% TFA, B = MeCN with 0.1% TFA; flow rate: 1.0 mL/min; gradient conditions: 0 min, 5% B; 0–10 min, 5→70% B; 10–12 min, 70% B; 12–15 min, 70→5% B. Unless specified, all ^225^Ac samples were counted (CPM) at approximately 16 h after collection to allow reaching secular equilibrium with gamma-emitting progeny isotopes (e.g., ^213^Bi), in an automated gamma counter (Cobra II, Packard).

### Synthesis of nonradioactive Lu-PSMA-617 and Pb-PSMA-617 complexes

For the synthesis of nonradioactive reference standards, PSMA-617 (1 mg, 1 µmol) was reacted with either LuCl_3_ (0.6 mg, 1.4 µmol) in 0.25 M NaOAc buffer (pH 5.5, 150 µL) or PbCl_2_ (0.42 mg, 1.5 µmol, 1.5 equiv.) in 0.25 M NaOAc buffer (pH 5.5, 150 µL). The reaction mixture was heated at 90 °C for 30 min, and the resulting complexes, Lu-PSMA-617 and Pb-PSMA-617, were purified using an Oasis HLB Plus Light cartridge (30 mg sorbent, Waters), eluted with ethanol. The purified complexes were characterized using LC-MS. Lu-PSMA-617: white solid, isolated yield: 0.45 mg (37%). ESI-MS: calc. for C_49_H_69_LuN_9_O_16_ [M + H]⁺: 1214.4; found, [M + H]⁺: 1214.1. Pb-PSMA-617: white solid, isolated yield: 0.6 mg (48%). ESI-MS, calc. for C_49_H_69_N_9_O_16_Pb [M + H]⁺: 1247.4629, [M + 2H]^2^⁺: 624.8, found, [M + 2H]^2^⁺: 624.8.

### Radiochemistry

In a 1.5 mL protein Eppendorf tube (LoBind®), approximately 37.0 MBq of ^177^LuCl_3_ in 0.25 M NaOAc buffer (pH 5.5, 100 µL) or 18.5 MBq of ^212^PbCl_2_ in 0.2 M NaOAc buffer (pH 5.5, 100 µL) was mixed with PSMA-617 (1.0 nmol, 1.0 µL). The reaction mixture was heated at 90 °C for 30 min in a thermomixer (500 rpm) to obtain the corresponding radioligand. Radiolabeling efficiency was evaluated by radio-HPLC as described above. The reaction mixture was cooled to room temperature and purified using an Oasis HLB Plus Light cartridge (30 mg, Waters). The cartridge was washed with water (2 × 2 mL), and the radiolabeled product was eluted with ethanol (200 proof, 0.5 mL) into a vial for use in in vitro studies.

For ^225^Ac labeling of PSMA-617, ^225^AcCl_3_ (0.92 MBq) was added to 0.2 M NH_4_OAc buffer (pH 5.2, 100 µL) in a 0.5 mL Eppendorf tube (LoBind), followed by the addition of PSMA-617 (1.0 nmol, 1.0 µL). The reaction mixture was heated at 95 °C for 45 min in a thermomixer (500 rpm), after which time the reaction mixture was cooled to room temperature and quenched with 50 nM DTPA solution (4 µL). The crude mixture was purified using an Oasis HLB Plus Light cartridge (30 mg), washed with water (2 × 2 mL), and the labeled compound was eluted with ethanol (200 proof, 0.5 mL).

### Distribution Coefficient (***D***)

The distribution coefficient (*D*) of the PSMA-617 radioligands was evaluated by the shake-flask method. Briefly, an aliquot of the radioligands was added to a mixture of *n*-octanol and PBS pH 7.4 (2 mL each, *n* = 3–4) in polypropylene tubes and vortexed for 1 min. The octanol and aqueous layers were separated by centrifugation at 3000 rpm for 5 min. From each tube, 0.5 mL aliquots of octanol and water phases were carefully withdrawn into pre-weighed Eppendorf tubes and measured for radioactivity in an automated gamma counter (Cobra II, Packard). Samples were weighed and normalized for density to get radioactivity counts as CPM/mL. Distribution coefficient (*D*) values were calculated as the ratio of CPM/mL in the octanol phase to that in the aqueous phase and presented as log*D* (mean ± SD) for each radioligand.

### In vitro stability studies

In a 1.5 mL Eppendorf tube (LoBind®), approximately 3.7 MBq of [^177^Lu]Lu-PSMA-617, 74 kBq of [^225^Ac]Ac-PSMA-617, or 0.37 MBq of [^212^Pb]Pb-PSMA-617 were incubated with 0.2 mL of PBS (pH 7.4), human serum, or human whole blood at 37 °C for up to 5 days in a thermomixer (500 rpm). For [^212^Pb]Pb-PSMA-617, the incubation period was limited to 24 h due to its short physical half-life (10.6 h). At 24 h intervals, a 20 µL aliquot was withdrawn and analyzed by iTLC. The strips were developed in 0.1 M citrate buffer (pH 7.4), dried, cut into 1 cm sections, and measured for radioactivity using an automated gamma counter (Cobra II, Packard). For [^225^Ac]Ac-PSMA-617, the iTLC strips were recounted after at least 16 h to allow secular equilibrium of ɣ-emitting progeny isotopes (e.g., ^213^Bi).

### Immunocytochemistry studies

For immunocytochemistry, 1 × 10^6^ cells were seeded in 6-well plates containing poly-L-lysine-coated coverslips (Fisher Scientific) and allowed to attach before staining. Cells were fixed with 2% methanol-free formaldehyde prepared in 1x PBS for 15 min at room temperature and washed three times with PBS. Cells were then permeabilized by incubation with methanol at -20 °C for 15 min and blocked for 1 h at room temperature in PBS supplemented with 5% normal serum (Cell Signaling Technology) and 0.1% Triton X-100. The cells were incubated overnight at 4 °C with primary antibody against PSMA (Cell Signaling Technology, catalog #12702) diluted 1:400 in PBS containing 0.1% Triton X-100. Following primary antibody incubation, samples were washed three times with PBS and incubated for 1 h at room temperature protected from light with Alexa Fluor 550-conjugated anti-rabbit IgG (H + L) F(ab’)_2_ fragment secondary antibody (Cell Signaling Technology) diluted 1:1000 in PBS containing 0.1% Triton X-100. Cells were washed three times with PBS and mounted using ProLong Diamond Antifade Mountant with DAPI (Invitrogen, #P36971). Fluorescence and bright-field images were acquired using an EVOS fluorescence microscope at 40× magnification.

### Cell uptake and internalization studies

LNCaP-AR, CWRR1-EnzR, DU145-PSMA, and DU145 cells were cultured in RPMI-1640 medium (ATCC) supplemented with 10% FBS (Corning) and 1% penicillin/streptomycin, with the additional supplements for the following cell lines: 1% HEPES, 1% sodium pyruvate, 1% Glutamax for LNCaP-AR; 10 µM enzalutamide (MedChemExpress) for CWRR1-EnzR. For cell uptake and internalization studies, cells were seeded in 24-well plates at 150,000 cells per well in triplicate and allowed to adhere overnight in the growth medium. On the following day, the culture medium was removed, and cells were washed once with ice-cold PBS. Cells were then incubated at 37 °C with 74 kBq of [^177^Lu]Lu-PSMA-617, 7.4 kBq of [^225^Ac]Ac-PSMA-617, or 18.5 kBq of [^212^Pb]Pb-PSMA-617 in 0.5 mL serum-free medium, either in the presence or absence of an excess of unlabeled PSMA-617 (300 nM) to assess blocking. Unless specified, the cell uptake studies of [^225^Ac]Ac-PSMA-617 were conducted in the presence of DTPA (0.1 mg/mL) in the incubation medium. At predetermined time points (0.5, 1, 2, and 4 h), the supernatant was collected, and cells were washed twice with 0.5 mL ice-cold PBS, collecting washes. Next, cells were incubated with 0.5 mL of ice-cold glycine-HCl buffer (50 mM, pH 2.5-3.0) for 5 min at room temperature to isolate cell surface-bound radioactivity, followed by an additional PBS wash. The glycine wash and PBS wash were pooled to represent the cell surface-bound fraction. Subsequently, cells were lysed using 0.25 mL Cell Lysis Reagent (Promega), and the wells were rinsed with 0.25 mL PBS, which was combined with the lysis fraction to obtain the internalized fraction. Radioactivity counts (CPM) in all fractions were measured using an automated gamma counter (Cobra II). Cell uptake was calculated as the percentage of the input dose (CPM) present in the cell fraction for each well and expressed as mean ± standard deviation (SD) for triplicate wells at each time point. Cell surface-bound (glycine wash) and internalized (lysis fraction) fractions were also determined as percentages of the initially bound activity to assess internalization rate for each radioligand at different time points.

### Inhibitory potency (IC_50_) assays

The inhibitory potencies of the nonradioactive Lu-PSMA-617 and Pb-PSMA-617 analogues were determined by competitive inhibition assays using [^177^Lu]Lu-PSMA-617 in the DU145-PSMA cell line. Cells seeded in 24-well plates (150,000 cells/well) were incubated with [^177^Lu]Lu-PSMA-617 (74 kBq/well) in serum-free medium for 2 h under standard cell culture conditions in the presence of increasing concentrations of Lu-PSMA-617 or Pb-PSMA-617 (0.14–300 nM, 3-fold serial dilutions, *n* = 8). After incubation, the medium was collected, and cells were washed with cold PBS (2 × 0.5 mL). Cells were then lysed with 1× Cell Lysis Reagent (0.25 mL, Promega), followed by an additional PBS wash (0.25 mL). Radioactivity in each fraction was measured using an automated gamma counter. Cell-bound activity was determined by calculating the percentage of the input dose (CPM). Half-maximal inhibitory concentration values (IC_50_) for the two nonradioactive conjugates were determined by nonlinear regression analysis of the inhibitory data using a log(inhibitor) versus response model in GraphPad Prism (v10.6).

### Saturation binding assays

The binding affinities (*K*_D_) of the PSMA radioligands were determined by saturation binding assays using the two PSMA-positive cell lines LNCaP-AR and DU145-PSMA. Cells seeded in 24-well plates (150,000 cells/well) were incubated with [^177^Lu]Lu-PSMA-617, [^225^Ac]Ac-PSMA-617, or [^212^Pb]Pb-PSMA-617 for 2 h at 37 °C in serum-free medium containing a series of increasing concentrations of the corresponding radioligand (0.40–50 nM, *n* = 8), prepared by two-fold serial dilutions starting from 50 nM. For each concentration, nonspecific binding was assessed in parallel by co-incubation with an excess of unlabeled PSMA-617 (300 nM). After incubation, the supernatant was collected, and the cells were washed with ice-cold PBS and lysed as described for cell uptake studies. Radioactivity (CPM) in the cell fractions was measured using an automated gamma counter (Cobra II). The equilibrium dissociation constant (*K*_D_) was calculated by nonlinear regression analysis of the data in GraphPad Prism (v10.6).

## Supplementary Information

Below is the link to the electronic supplementary material.

Supplementary Material 1

## Data Availability

The data is available upon request.

## Abbreviations

PSMA: prostate-specific membrane antigen
RLT: radioligand therapy
LET: linear energy transfer
Lu: lutetium
Ac: actinium
Pb: lead
Bi: bismuth
kBq: kilobecquerel
MBq: megabecquerel
nmol: nanomoles
M: molar
nM: nanomolar
µM: micromolar
µmol: micromole
PBS: phosphate-buffered saline
K_D_: equilibrium dissociation constant
mCRPC: metastatic castration-resistant prostate cancer
keV: kiloelectronvolt
µm: micrometer
DNA: deoxyribonucleic acid
HPLC: high-performance liquid chromatography
LC-MS: liquid chromatography-mass spectrometry
iTLC: instant thin-layer chromatography
D: distribution coefficient
CPM: counts per minute
h: hours
min: minutes
%AA: percent added activity
DTPA: diethylenetriaminepentaacetic acid
mg: milligram
mL: milliliter
µL: microliter
α: alpha
β: beta
γ: gamma
QC: quality control
RCY: radiochemical yield
HCl: hydrochloric acid
NaOAc: sodium acetate
NH_4_OAc: ammonium acetate
ESI-MS: electrospray ionization mass spectrometry
MeCN: acetonitrile
TFA: trifluoroacetic acid
Å: angstrom
