# Supplementary material for: Radiochemistry and comparative in vitro assessment of PSMA-617 labeled with lead-212 (212Pb), actinium-225 (225Ac), and lutetium-177 (177Lu)
Source: EJNMMI Radiopharm Chem. 2026 May 20;11:42. doi: 10.1186/s41181-026-00456-w (PMC13328690; doi:10.1186/s41181-026-00456-w)
Supplement: Supplementary file 1 — Supplementary Material 1 [file 41181_2026_456_MOESM1_ESM.docx]

**Supplementary Information**

**Radiochemistry and Comparative In Vitro Assessment of PSMA-617 Labeled with Lead-212 (^212^Pb), Actinium-225 (^225^Ac), and Lutetium-177 (^177^Lu).**

Abhijit Bera^1^, Graham Ragland^1^, Yuhan Zhang^2^, Patricia G. Madel^2^, Jasmine B'Lanton^2^, Atchimnaidu Siriki^1^, Chin-Tu Chen^1^, Russell Z. Szmulewitz^2^, Satish K. Chitneni^1*^

^1^Department of Radiology and ^2^Department of Medicine, The University of Chicago, Chicago, IL, USA

^*^Correspondence: [schitneni@uchicago.edu](mailto:schitneni@uchicago.edu)

**Table of Contents**

Synthesis scheme and LC-MS analysis of nonradioactive Lu-PSMA-617…………………………p2

Synthesis scheme and LC-MS analysis of nonradioactive Pb-PSMA-617 ……………………….p3

Radiolabeling and QC analysis of [^177^Lu]Lu-PSMA-617…………………………………………….p4

Radiolabeling and QC analysis of [^225^Ac]Ac-PSMA-617………………….………………………….p5

Radiolabeling and QC analysis of [^212^Pb]Pb-PSMA-617………………...……………………….....p6

Stability profiles of [^177^Lu]Lu-PSMA-617, [^225^Ac]Ac-PSMA-617 and [^212^Pb]Pb-PSMA-617……….p7

Cell uptake of the three radioligands in LNCaP-AR and DU145-PSMA cell lines………….………p8

Binding affinity assay of [^225^Ac]Ac-PSMA-617 without DTPA in the incubation medium………....p9

**A**


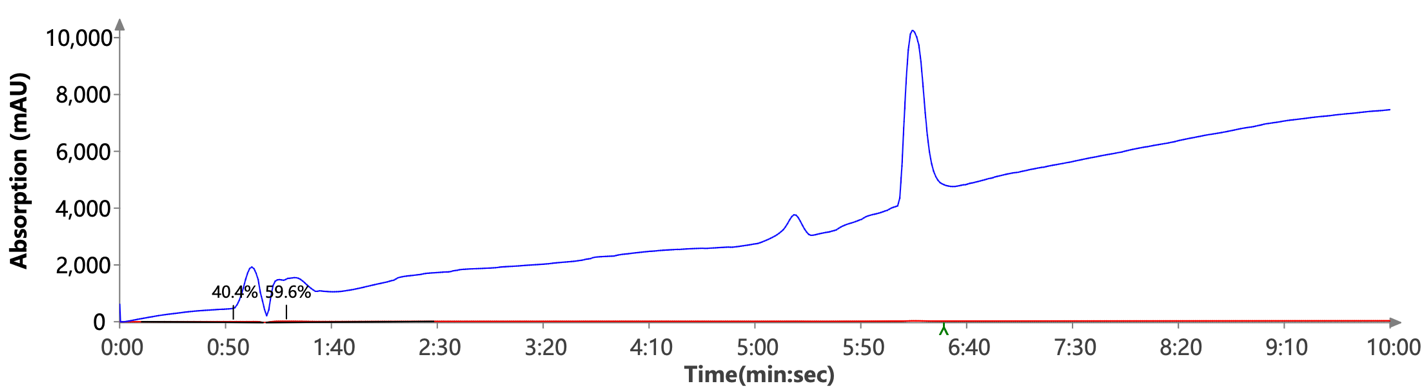

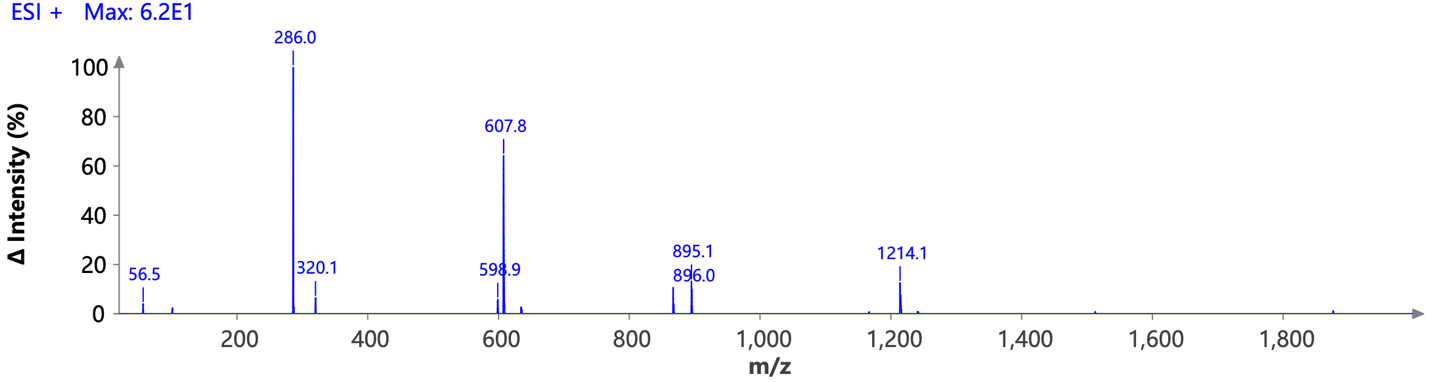


**B**

**Figure S1.** (**A**) Synthesis scheme for the nonradioactive Lu-PSMA-617. (**B**) LC-MS analysis of the purified Lu-PSMA-617 (*m/z*: 1214.1 [M+H]^+^).

**A**


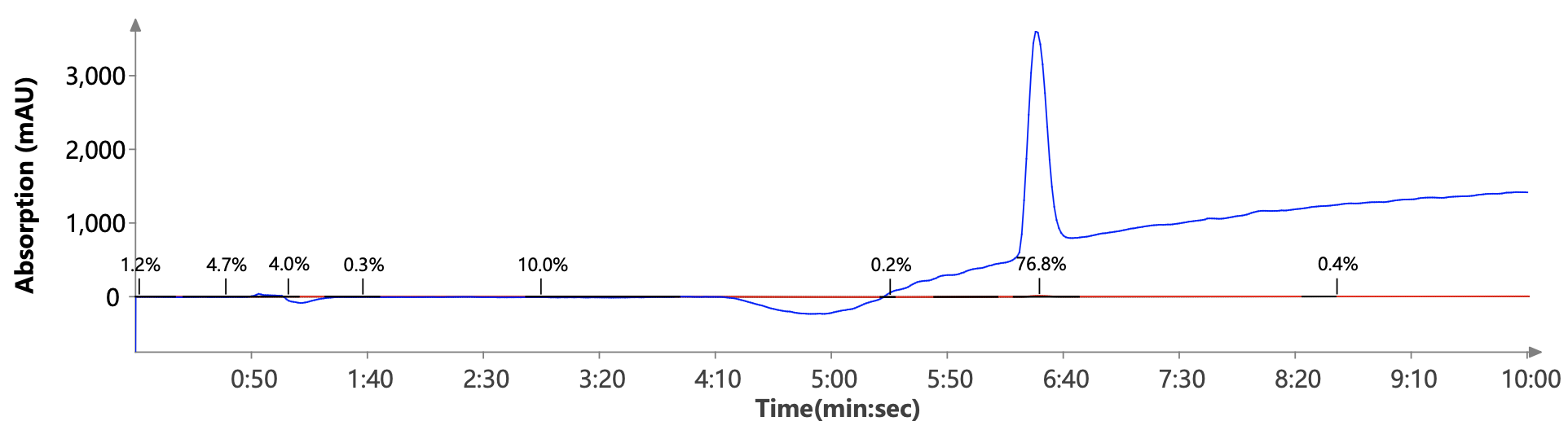

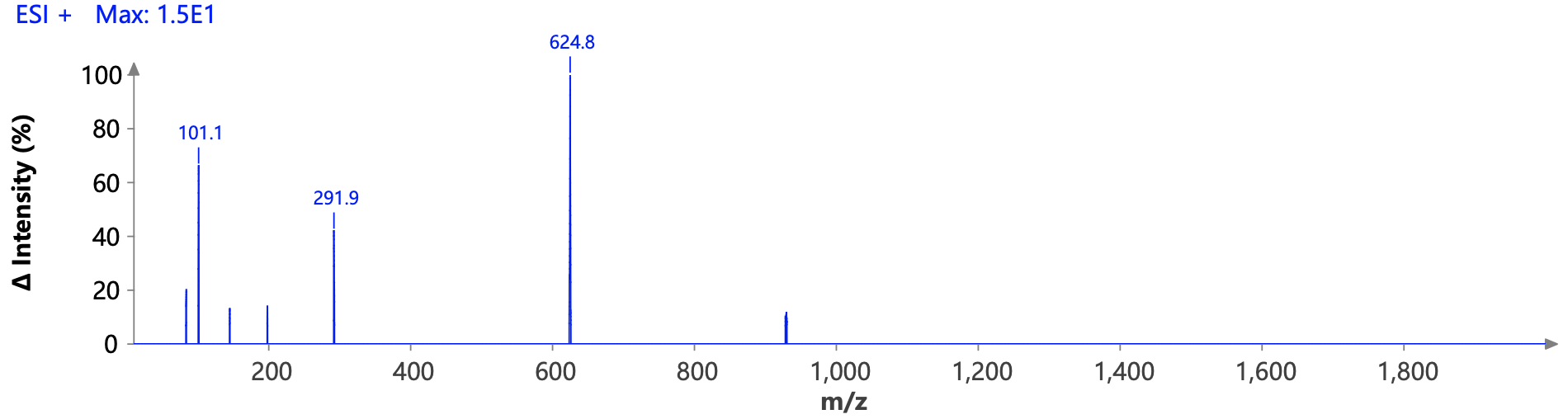


**B**

**Figure S2.** (**A**) Synthesis scheme for the nonradioactive Pb-PSMA-617. (**B**) LC-MS analysis of the purified Pb-PSMA-617 (*m/z*: 1247.46 ([M+H]^+^), *m/z*: 624.8 ([M+2H]^+2^)).

**A**

**B**

**C**

[^177^Lu]Lu-PSMA-617

[^177^Lu]Lu-PSMA-617

Free ^177^Lu

**Figure S3.** (**A**) Radiolabeling of PSMA-617 with ^177^Lu. (**B**) Radio-HPLC chromatogram of the crude labeling mixture of [^177^Lu]Lu-PSMA-617. (**C**) Radio-HPLC chromatogram of [^177^Lu]Lu-PSMA-617 after Sep-Pak purification.

**A**


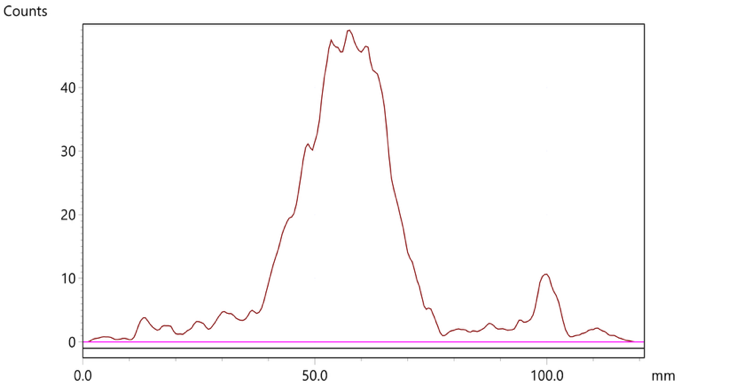


**B**

[^225^Ac]Ac-PSMA-617

Free ^225^Ac


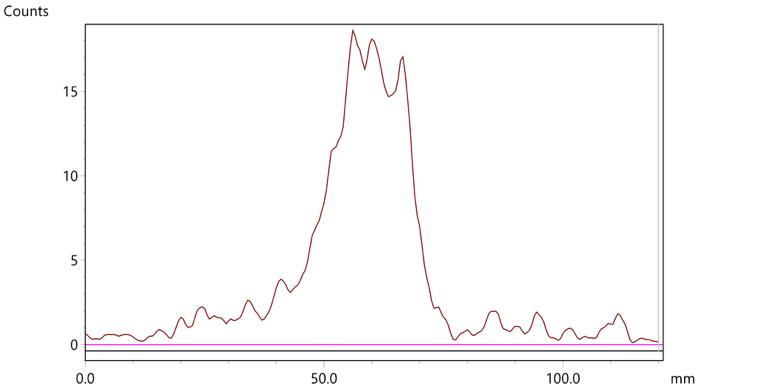


**C**

[^225^Ac]Ac-PSMA-617

**Figure S4.** (**A**) Radiolabeling of PSMA-617 with ^225^Ac. (**B**) iTLC chromatogram of the crude labeling mixture of [^225^Ac]Ac-PSMA-617. (**C**) iTLC chromatogram of [^225^Ac]Ac-PSMA-617 after Sep-Pak purification.

**B**

Free ^212^Pb

[^212^Pb]Pb-PSMA-617

**C**

[^212^Pb]Pb-PSMA-617

**A**

**Figure S5.** (**A**) Radiolabeling of PSMA-617 with ^212^Pb. (**B**) Radio-HPLC chromatogram of the crude labeling mixture of [^212^Pb]Pb-PSMA-617. (**C**) Radio-HPLC chromatogram of [^212^Pb]Pb-PSMA-617 after Sep-Pak purification.

**A**

**B**

**C**

**Figure S6.** In vitro stability of [^177^Lu]Lu-PSMA-617 (**A**), [^225^Ac]Ac-PSMA-617 (**B**), and [^212^Pb]Pb-PSMA-617 (**C**) in PBS, human serum, and human whole blood.

**A**

**B**

**Figure S7.** Comparison of the cell uptake of [^177^Lu]Lu-PSMA-617, [^212^Pb]Pb-PSMA-617, and [^225^Ac]Ac-PSMA-617, in the PSMA-positive LNCaP-AR cell line (**A**), and in the PSMA-overexpressing DU145-PSMA cell line (**B**).

**Figure S8**. Saturation binding assay of [^225^Ac]Ac-PSMA-617 in the PSMA-positive LNCaP-AR cell line with no DTPA in the incubation medium.

**Scheme 3.** Radiolabeling of PSMA-617 with ^225^Ac.


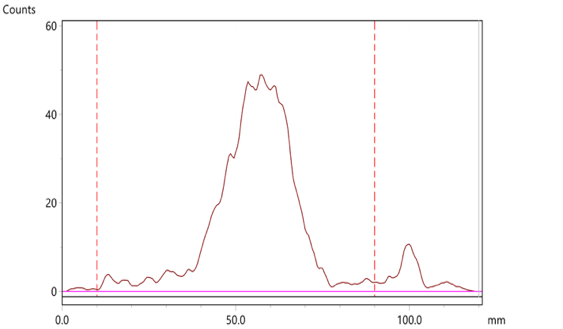

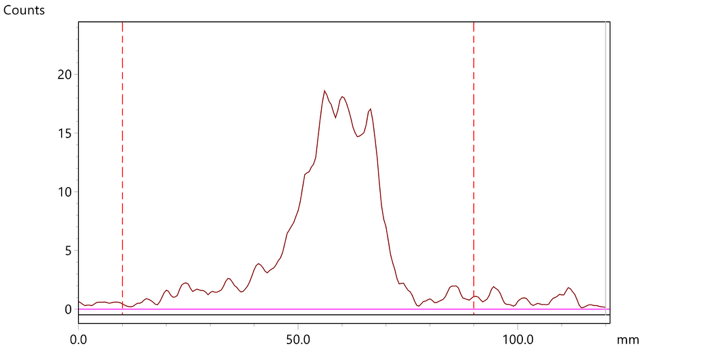


**A**

**B**

^225^Ac-PSMA-617

**Fig. 7.** iTLC analysis of the ^225^Ac-PSMA-617 crude reaction mixture (A) and after purification with a Sep-Pak (B). Analysis was done immediately after the reaction.

^225^Ac

^225^Ac-PSMA-617
